# Assessing the effects of mitofusin 2 deficiency in the adult heart using 3D electron tomography

**DOI:** 10.14814/phy2.13437

**Published:** 2017-09-14

**Authors:** Siavash Beikoghli Kalkhoran, Andrew R. Hall, Ian J. White, Jackie Cooper, Qiao Fan, Sang‐Bing Ong, Sauri Hernández‐Reséndiz, Hector Cabrera‐Fuentes, Kroekkiat Chinda, Bibhas Chakraborty, Gerald W. Dorn, Derek M. Yellon, Derek J. Hausenloy

**Affiliations:** ^1^ The Hatter Cardiovascular Institute University College London London United Kingdom; ^2^ The National Institute of Health Research University College London Hospitals Biomedical Research Centre London United Kingdom; ^3^ Institute of Cardiovascular Science University College London London United Kingdom; ^4^ MRC Laboratory of Molecular Cell Biology University College London London United Kingdom; ^5^ Centre for Quantitative Medicine Duke‐NUS Medical School Singapore; ^6^ Cardiovascular and Metabolic Disorder Programme Duke‐NUS Medical School Singapore; ^7^ National Heart Research Institute Singapore National Heart Centre Singapore Singapore; ^8^ Department of Physiology Faculty of Medical Science Naresuan University Phitsanulok Thailand; ^9^ Centre for Pharmacogenomics Department of Internal Medicine Washington University School of Medicine St. Louis Missouri; ^10^ Barts Heart Centre St Bartholomew's Hospital London United Kingdom; ^11^ Yong Loo Lin School of Medicine National University Singapore Singapore

**Keywords:** 3D electron tomography, MFN2 KO, mitochondria–jSR junction

## Abstract

The effects of mitofusin 2 (MFN2) deficiency, on mitochondrial morphology and the mitochondria–junctional sarcoplasmic reticulum (jSR) complex in the adult heart, have been previously investigated using 2D electron microscopy, an approach which is unable to provide a 3D spatial assessment of these imaging parameters. Here, we use 3D electron tomography to show that MFN2‐deficient mitochondria are larger in volume, more elongated, and less rounded; have fewer mitochondria–jSR contacts, and an increase in the distance between mitochondria and jSR, when compared to WT mitochondria. In comparison to 2D electron microscopy, 3D electron tomography can provide further insights into mitochondrial morphology and the mitochondria–jSR complex in the adult heart.

## Introduction

Changes in mitochondrial morphology through the processes of fusion and fission affect cellular function and survival (Gomes et al. 2011; Ong et al. 2013). The importance of these morphological alterations can be highlighted by active participation of fission and fusion in cellular differentiation during embryonic development, adaptation to stress, organelle quality control through autophagy, and the induction of cell death (Frank et al. 2001; Twig et al. 2008; Mishra et al. 2014; Song et al. 2015). The mitochondrial fusion proteins, mitofusin 1 and 2 and OPA1, mediate fusion of the mitochondrial outer and inner membranes, respectively, generating elongated interconnected mitochondria, whereas the mitochondrial fission proteins, Drp1, hFis1, MFF, and MiD49/51, induce fragmented disconnected mitochondria (Hall et al. 2014). Investigating the role of the mitochondrial fusion and fission proteins on mitochondrial morphology in the adult heart, in which mitochondria are characteristically arranged in rigid networks aligned with the myofibrils, can be quite challenging using current experimental techniques: 2D electron microscopy (EM) is limited to one single 2D plane and can only provide an approximation of mitochondrial shape and size in one planar axis (Lewis et al. 1999; Picard et al. 2013; Neary et al. 2014), confocal microscopy has limited image resolution (200 *μ*m in the *x–y* axis) (Mariotti et al. 2015), and flow cytometry can only assess the morphology of isolated mitochondria (Cottet‐Rousselle et al. 2011).

Furthermore, these techniques are unable to accurately assess the spatial interaction of mitochondria with SR, a spatial relationship which is essential to facilitate calcium signaling between these two organelles, and which couples mitochondrial energy production with contraction in the adult heart (de Brito and Scorrano 2008; Korobova et al. 2013). MFN2 is one of the central players in the field of cardiac energetics and the deficiency in the function of this protein induces the Charcot Marie tooth disease (Cartoni and Martinou 2009; Chen et al. 2012b). In addition to its profusion effect, MFN2 has also been shown to tether mitochondria to SR, and derangements in this interaction in hearts deficient in MFN2 have been shown to affect calcium signaling between mitochondria and SR, endoplasmic reticulum stress, mitochondrial respiration, and cardiomyocyte contractile function (Papanicolaou et al. 2011; Chen et al. 2012b; Ngoh et al. 2012; Chen and Dorn 2013). Until now, studies have used 2D EM to assess the spatial relationship between mitochondria and junctional SR (jSR) (Boncompagni et al. 2009; García‐Pérez et al. 2011), but this approach does not take into account the 3D spatial relationship between these two organelles. Therefore, in this study we use 3D electron tomography to assess mitochondrial morphology and the interaction of mitochondria with junctional SR in adult hearts deficient in MFN2.

## Methods

### Mouse strains/genotyping

All animal experiments were performed in compliance with the Animals (Scientific Procedures) Act 1986 published by the U.K. Home Office. We used transgenic mice in which cardiomyocyte‐specific gene deletion of MFN2 occurs soon after birth (MFN2 loxp/loxp mice crossed onto cardiac‐specific Myh6 nuclear‐directed “turbo” Cre) (Chen et al. 2003, 2007, 2012b). Female MFN2 KO and wild‐type (WT) littermate mice at 8–10 weeks were used for the preparation of all heart samples. To determine the genotype of each individual animal, their ear snips were obtained and lysed using DirectPCR Lysis Reagent (402‐E/Viagen). The PCR was performed using the Taq DNA Polymerase (Qiagen‐201203) using reverse and forward primers targeted against the Cre gene. PCR samples were then transferred to a 2% agarose gel containing Syto60 (1: 100,000‐Invitrogen/S11342) and were imaged using the Odyssey imaging system from Li‐Cor Biosciences. The genotyping was based on the Cre expression in individual animals as shown in Figure S1. All materials were purchased from Sigma.

### Transmission electron microscopy

WT (*N* = 5) and MFN2 KO hearts (*N* = 5) were initially extracted and fixed using EM grade 1% paraformaldehyde, 2% glutaraldehyde in 0.1 mol/L sodium cacodylate buffer. Transverse sections of the left ventricle were dissected from fixed hearts, and postfixed in 1% osmium tetroxide and 1.5% potassium ferricyanide for 1 h at 4°C before further staining with aqueous 2% uranyl acetate. Samples were sequentially dehydrated using increasing concentration of ethanol (25%, 50%, 70%, 90%, 100%) and washed with 1,2‐epoxypropane, before embedding in epon resin. For 3D reconstruction, regions of each heart with transverse fibers were sectioned and 40 consecutive 70‐nm sections were collected and further stained with lead citrate. Three representative areas containing intermyofibrillar (IMF) mitochondria were randomly selected in each section series, and imaged across the full 40‐section range (2.8 *μ*m total depth) using a Tecnai G2 Spirit transmission EM (TEM) from FEI, equipped with an Olympus‐SIS Morada CCD camera. Captured images had a pixel size of 2.86 nm in both *x* and *y* axes. Video reconstruction was performed on images with the pixel resolution of 2.06 nm in both *x* and *y* axes.

### 3D image processing and data acquisition

**Figure 1:**
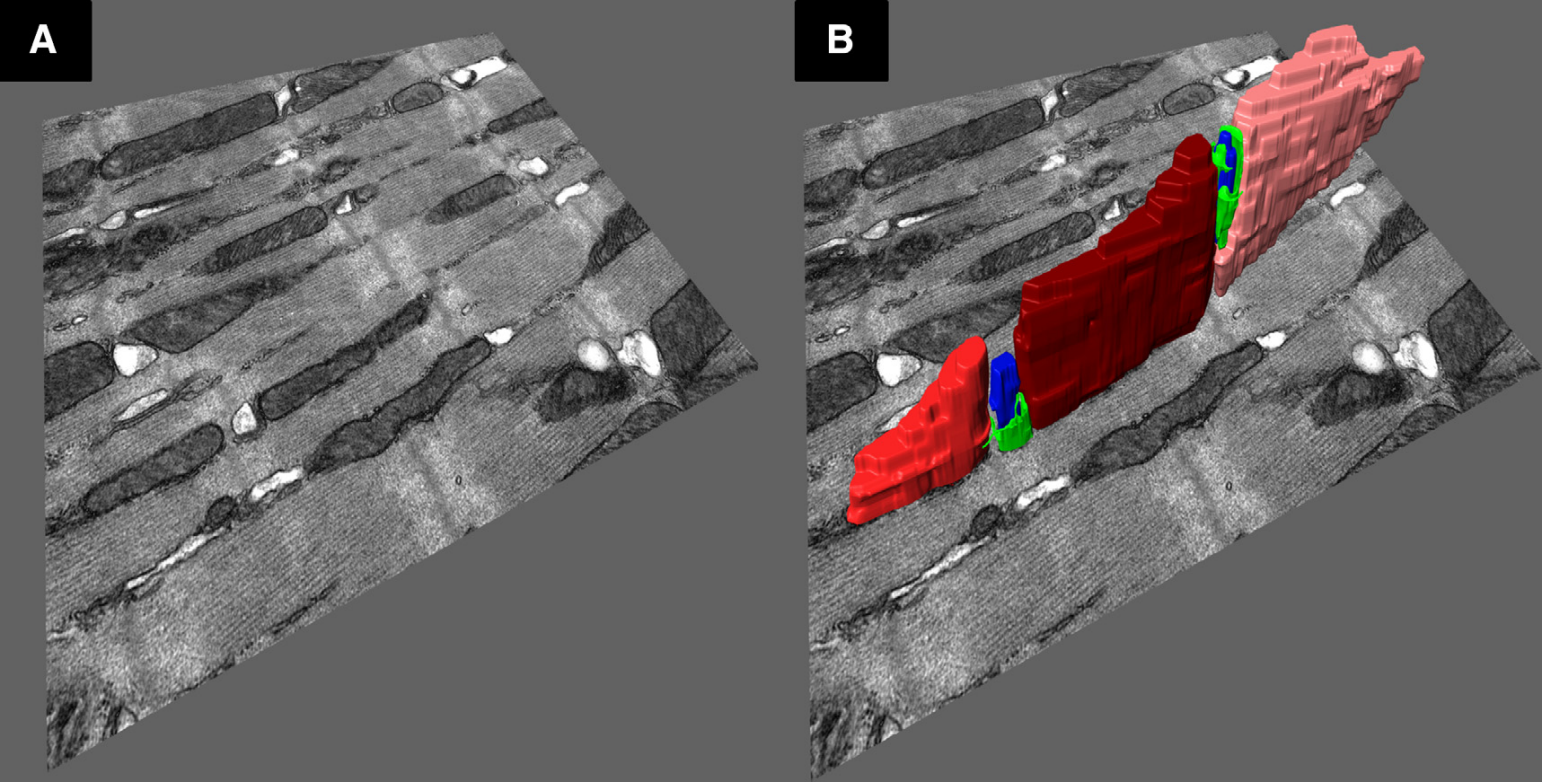
3D reconstruction of adult cardiac mitochondria and adjacent SR network. (A) TEM depicts intermyofibrillar mitochondria and adjacent SR network in adult mouse WT heart. (B) The 3D reconstruction of the jSR and T‐tubule network (shown by blue and green color, respectively) as well as their neighboring mitochondria (shown by different intensity of red color).

In total, 317 WT and 219 MFN2 KO cardiac IMF mitochondria were reconstructed and analyzed in a blinded fashion. 3D reconstruction and quantification were performed using Amira 5.4.3. Sections were initially aligned and mitochondria were traced within each of the 40 sections. Subsequently, mitochondria were manually segmented, reconstructed (Fig. 1 and Movies S1 and S2), and their morphometric parameters were obtained using the multicomponent module of the Amira software. The multicomponent module is based on principal component analysis and allows the quantification of several imaging parameters as follows:

1. *Volume*: reported as “the volume of the region in units of the voxel size.”
2. *Anisotropy* (*roundness*): computed as “1 minus the ratio of the smallest to the largest eigenvalue of the covariance matrix. This parameter measures a region's deviation from a rounded shape.”
3. *Elongation*: computed as “the ratio of the medium and the largest eigenvalue of the covariance matrix” with value close to zero representing more elongated objects.
4. *Flatness*: defined as “the ratio of the smallest and the medium eigenvalue of the covariance matrix. Flat objects have small values close to 0.”

**Figure 2:**
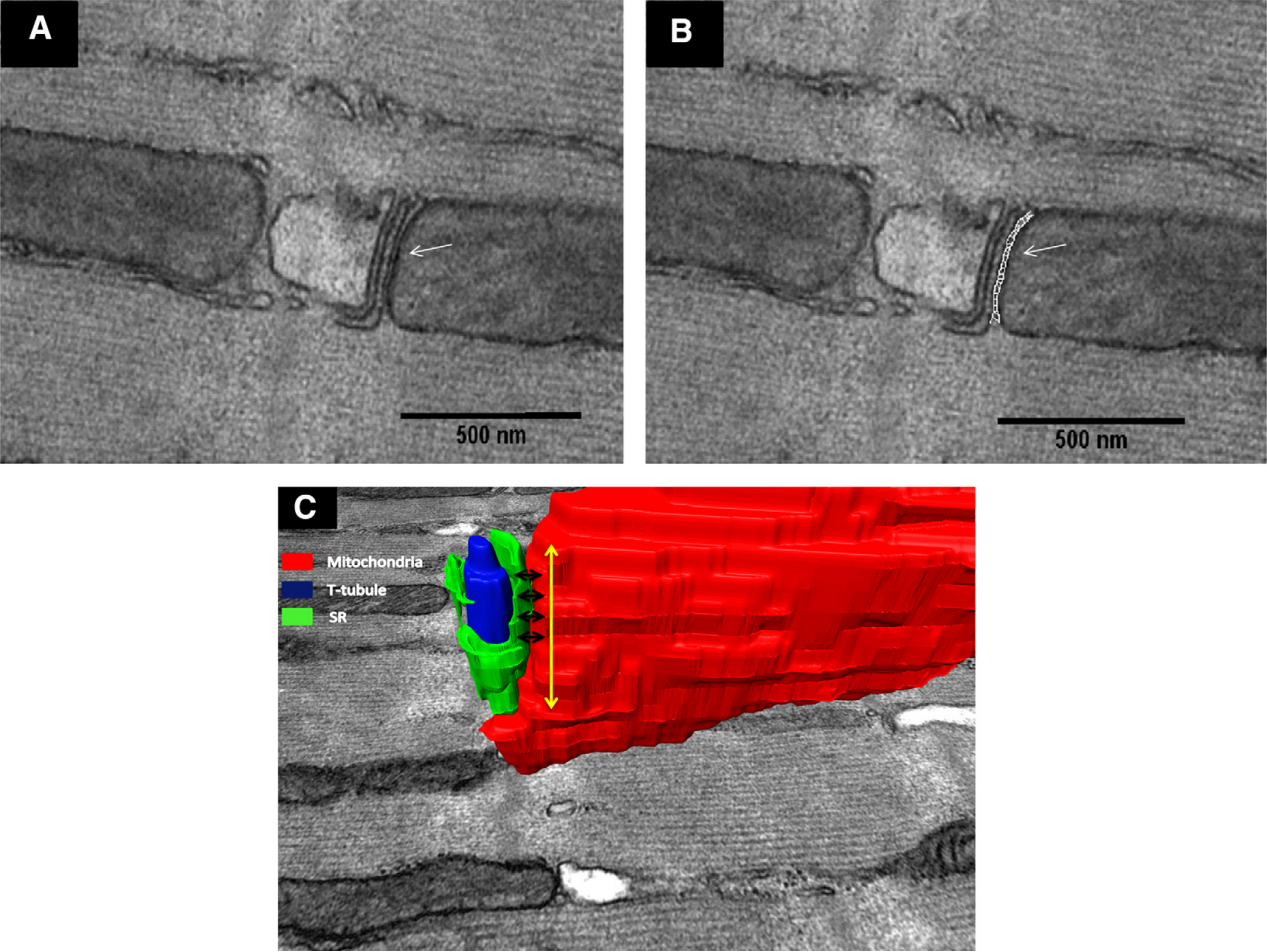
Measuring the minimum mitochondria–jSR distance. (A) TEM micrograph depicts spatial interaction between intermyofibrillar mitochondria and adjacent jSR and T‐tubule network in adult mouse WT heart (white arrow indicates mitochondria–jSR space without measurement). (B) The perpendicular lines that were drawn between the jSR and one mitochondrion to measure the minimum distance between these two organelles (white arrow indicates the perpendicular lines drawn on mitochondria–jSR space). (C) 3D reconstruction depicts the minimum distances (black arrows) between the mitochondrion (red) and jSR (green) along the interface between these two organelles (yellow arrow). The T‐tubule is represented in blue.

The minimum mean distance between mitochondria and jSR was measured using an image J (V. 1.46r) “ucl_2LineDist” plug‐in, which was a kind gift from Mr. Daniel Ciantar. For each 2D micrograph, two lines were manually drawn on the surface of jSR and mitochondria. The plug‐in was then used to estimate the minimum distance between the two organelles by automatically drawing at least 20 perpendicular lines between the two organelles (Fig. 2A and B). This process was repeated along the *z*‐axis for the same network to obtain the mean of the 3D distance between the two organelles. Distances above 50 nm were excluded from the analysis (Chen et al. 2012b). Additionally, the mean length of the spread of network along the *z*‐axis was measured via multiplication of the number of sections and slice thickness for each individual network (Fig. 2C).

### Statistical analysis

Statistical tests were performed using Stata (v.13). Nested mixed effects multilevel regression was used to compare the morphological parameters between the two different genotypes. This technique accounts for cell‐to‐cell and heart‐to‐heart variability within the data. The predicted means and standard errors from this model were used to compare individual shape descriptors of different genotypes. Spearman's rank correlation (denoted by “rho”) was used to plot the degree of correlation between each individual mitochondrial parameter with rho = 1 illustrating perfect positive correlation and rho = −1 indicating perfect negative correlation. Similar to the morphometric analysis, mixed effects multilevel regression was used to assess the mean distance and length in *z* direction between mitochondria and jSR to address the variability between the cells and the hearts. Predicted means and 95% confidence intervals were constructed using Microsoft Excel 2010 to show the differences between mitochondrial morphology and mitochondria–jSR interaction in WT and MFN2 KO hearts. For all of the mentioned statistical tests, a *P* ≤ 0.05 was considered significant.

## Results

### Morphological differences between MFN2 and WT mitochondria

**Figure 3:**
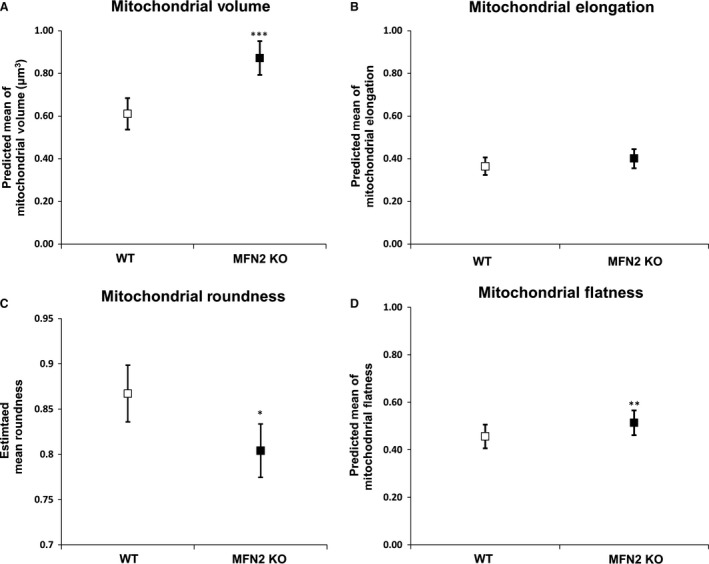
Morphometric parameters of WT and MFN2 KO cardiac mitochondria. This figure shows that MFN2 KO mitochondria have increased 3D volume (A), are equally elongated (B), are less rounded (C), and are flatter (D) when compared to WT mitochondria. Values are mean and confidence interval (CI); 317 WT and 219 MFN2 KO mitochondria (*N* = 5 WT and MFN2 KO mice). (*, **, and *** denote *P* ≤ 0.05, *P* < 0.001, and *P* < 0.0001, respectively).

MFN2 KO mitochondria, when compared to WT mitochondria, had greater volume (0.87 ± 0.08 *μ*m^3^ MFN2 KO vs. 0.61 ± 0.07 *μ*m^3^ WT, *P* < 0.001; Fig. 3A and Fig. S2A) and flatness (0.51 ± 0.03 MFN2 KO vs. 0.46 ± 0.03 WT, *P* < 0.001; Fig. 3D and Fig. S2D) while being almost equally elongated (0.40 ± 0.02 MFN2 KO vs. 0.36 ± 0.02 WT, *P* < 0.080; Fig. 3B and Fig. S2B) and less rounded in shape (0.80 ± 0.02 MFN2 KO vs. 0.83 ± 0.02 WT, *P* < 0.021; Fig. 3C and Fig. S2B). The alteration of some of these parameters can also be observed in Movies S1 and S2.

### Evaluation and correlation of mitochondrial morphology descriptors in 3D

**Figure 4:**
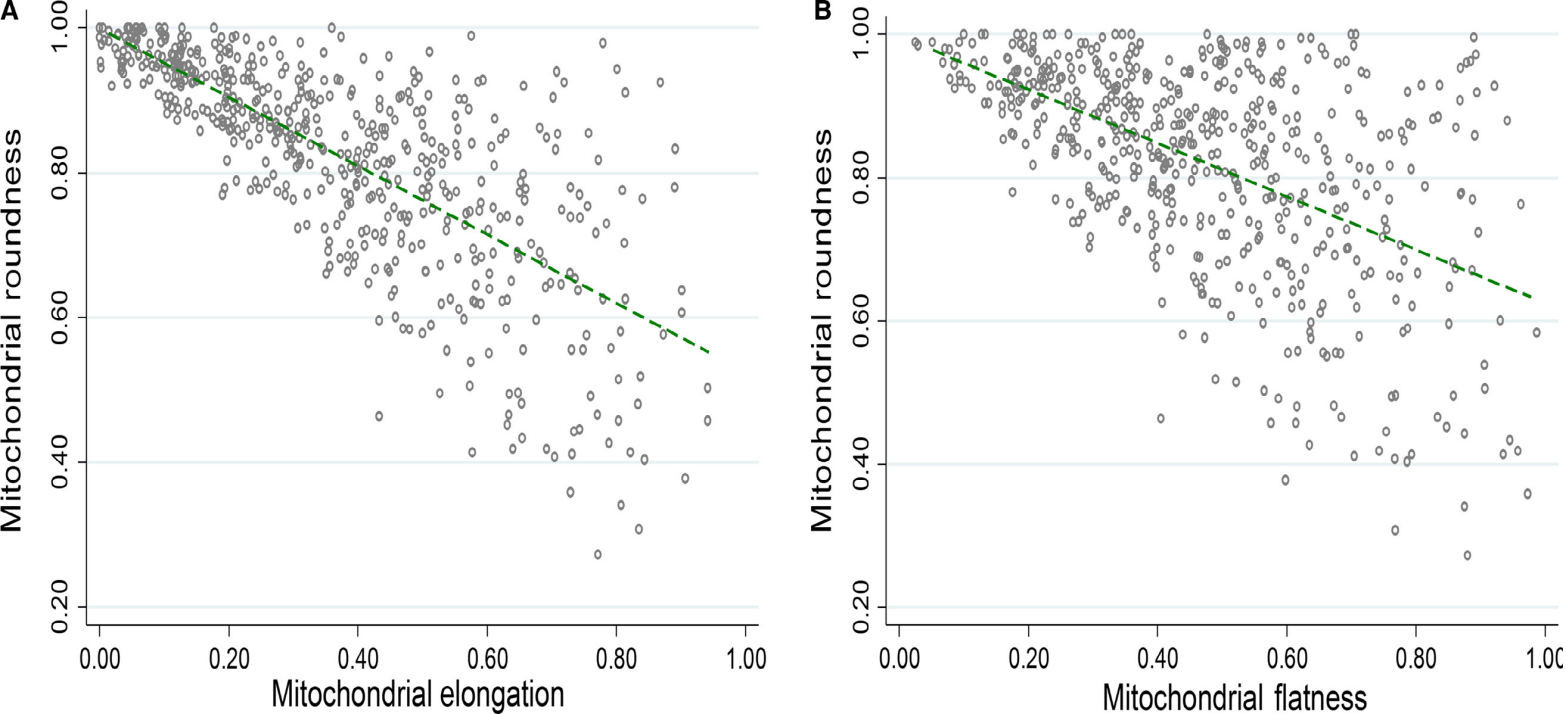
Correlation between mitochondrial morphometric parameters. There was a negative correlation between (A) elongation and roundness and (B) flatness and roundness; 317 WT and 219 MFN2 KO mitochondria (*N* = 5 WT and MFN2 KO mice).

To evaluate the impact of mitochondrial volume on other shape summaries, we looked at the correlation between different mitochondrial parameters in 3D. Mitochondrial volume was not significantly associated with any of the other three parameters (rho [*P* value]: 0.0007 [0.986] for flatness, −0.0359 [0.406] for roundness, and 0.0551 [0.202] for elongation). Mitochondrial roundness decreased significantly as both elongation (rho [*P* value]: −0.795 [0.0001]) and flatness (−0.5142 [0.0001]) increased as shown in Figure 4A and B, respectively. Besides, there was no correlation between elongation and flatness (rho [*P* value]: −0.0260 [0.547]). Hence, mitochondrial roundness is correlated with flatness and elongation, whereas the physical size of the mitochondria does not influence these parameters.

### Mitochondria–jSR distance assessed by 3D electron tomography

**Figure 5:**
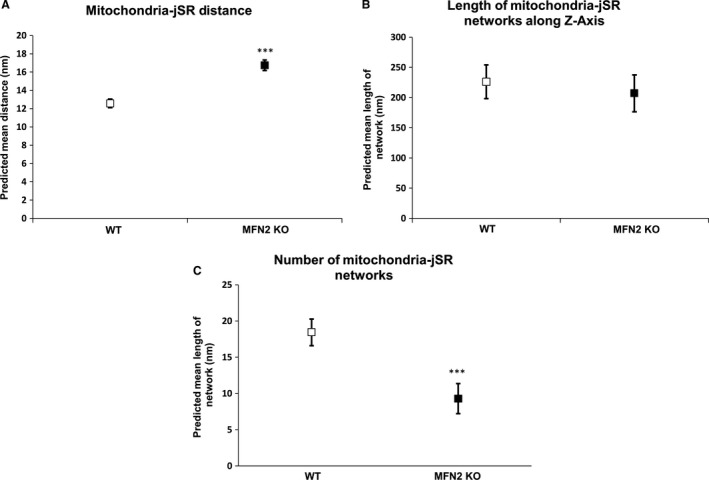
Mitochondria–jSR interaction in WT and MFN2 KO hearts. (A) There was an increase in the minimum distance between mitochondria and jSR in MFN2 KO heart when compared to WT hearts. (B) There was no significant difference in the length of the interface between mitochondria and jSR in WT or MFN2 KO hearts. (C) There were fewer mitochondria–jSR networks in MFN2 KO hearts when compared to WT ones (*N* = 5 WT and MFN2 KO mice).

Measuring the minimum distance between mitochondria and jSR indicated a significant increase in the distance between the two organelles (WT: 12.57 ± 0.24 nm vs. 16.75 ± 0.29 nm, *P* < 0.0001) (Fig. 5A). There was no substantial difference in terms of length of the interface between the two organelles (Fig. 5B) along *z*‐axis (WT: 226.24 nm ± 14.29 vs. 207.10 nm ± 15.63, *P* < 0.064). Intriguingly, the number of mitochondria–jSR networks were significantly lower in MFN2 KO in comparison to the WT hearts (WT: 18.45 ± 0.94 vs. 9.30 ± 1.06, *P* = 0.0001), see Figure 5C.

## Discussion

Using 3D electron tomography, we have shown that Mfn2‐deficient cardiac mitochondria are larger, flatter, and less rounded with regard to their 3D shape when compared to WT mitochondria. In addition, we have demonstrated that MFN2 KO hearts have fewer mitochondria–jSR networks and exhibit an increase in the minimum distance between mitochondria and jSR. These finding are consistent with the role of MFN2 as a tethering protein between mitochondria and jSR.

Investigating shape changes in mitochondria may help elucidate their dynamic behavior in cardiac health and disease. Deficiencies in proteins which regulate the shape of cardiac mitochondria induce a range of cardiac pathologies, including susceptibility to ischemia/reperfusion injury, dilated cardiomyopathy, heart failure, left ventricular hypertrophy, and vascular smooth muscle cell‐induced atherosclerosis (Zhou et al. 2010; Chen et al. 2011, 2012a; Papanicolaou et al. 2012; Ikeda et al. 2015; Lim et al. 2015; Hall et al. 2016), highlighting the importance of mitochondrial morphology in the normal functioning of the adult heart. In spite of being a 3D structure, organelles such as mitochondria are often quantified using 2D approaches, thereby ignoring their 3D structure (Boyce et al. 2010). 2D quantification is unable to accurately measure 3D morphometric parameters. Additionally, cardiac mitochondria extend along and across the myofibers and hence disparate parts of the same mitochondria may appear as different individual mitochondria in one single 2D plane. Therefore, 3D evaluation of mitochondrial shape can reaffirm the results of 2D analysis and rectify any bias that may rise from the 2D quantification of mitochondrial shape.

The mitochondrial morphometric parameters used in this study can provide insights into the overall morphology of individual adult cardiac mitochondria in 3D. The absence of any correlation between mitochondrial size and other shape descriptors indicates the sensitivity of these 3D shape descriptors to precisely define the actual morphology of mitochondria. Some of the correlative shape changes that we observed for 3D parameters are also present in 2D. For instance, the inverse correlation between mitochondrial roundness and elongation in our 3D model has been also described for the 2D model of IMF mitochondria from mouse skeletal muscle (Picard et al. 2013). Similarly, a decrease in the length of mitochondria in aged body wall muscles of the nematode *Caenorhabditis elegans* has been shown to be associated with an increase in the level of mitochondrial circularity (Regmi et al. 2014). Although these data may indicate the same pattern of changes for both 2D and 3D shape parameters, it is noteworthy to point that these tissues have a different mitochondrial architecture compared to the adult heart, hence rendering the 3D measurement as a more accurate methodology to investigate mitochondrial morphology in the adult heart.

Comparison of mitochondrial morphometric parameters in MFN2 KO and WT hearts showed a significant increase in mitochondrial elongation and volume. Similar to these findings, Walsh group observed an increase in the maximum and minimum diameter of mitochondria in MFN2 KO mice when compared to WT mice (Papanicolaou et al. 2011). The unusual increase in the 2D diameter of MFN2 KO mitochondria have been also described in skeletal muscle and neuronal cells; however, these mitochondria were suggested to be more rounded in comparison to the WT mitochondria (Chen et al. 2007, 2010). The measure of mitochondrial flatness which indicates the extent of branching of mitochondria was significantly decreased in MFN2 KO mice. This notion may contribute to the altered fusion dynamics observed in MFN2 KO skeletal muscle cells (Eisner et al. 2014). We also observed an enlargement in IMF mitochondria in MFN2 KO hearts that are consistent with flow cytometry analysis in previous studies (Papanicolaou et al. 2011; Song et al. 2014). The cause of the paradoxical increase in mitochondrial size in MFN2 KO hearts is not known and may be due to mitochondrial DNA damage in MFN2 KO hearts (Chen et al. 2014) or interruption of normal mitophagic mitochondrial turnover (Chen and Dorn 2013). In addition and in line with this fact, MFN2 also participates in the ER structure, the organelle which actively contributes to mitochondrial fission (de Brito and Scorrano 2008; Korobova et al. 2013). Nevertheless, the contribution of abrogated SR function and mitochondria DNA damage to the enlargement of MFN2 KO mitochondria remains inconclusive.

The mitochondrial fusion protein, MFN2, has been shown to act as a tether between mitochondria and ER in noncardiac cells, and hearts deficient in MFN2 have been shown to have an increased distance between jSR and mitochondria, assessed by 2D EM (de Brito and Scorrano 2008; Chen et al. 2012b). However, these quantification methods of the jSR–mitochondria interaction have been limited to the 2D image plane. As such, 3D electron tomography offers a more accurate assessment of the spatial relationship between mitochondrial and jSR. It is known that the synchronized interplay between SR and mitochondria in cardiomyocytes play a major role in cardiac cell Ca^2+^ signaling, phospholipid homeostasis, autophagy, and ROS generation (Eisner et al. 2013). This interplay is achieved by the presence of physical tethering between the two organelles and allows the formation of Ca^2+^ microdomains which are crucial for cardiac contractility (Dorn et al. 2015). The loss of tethering in MFN2‐deficient cardiac mitochondria affects the Ca^2+^ microdomain and induces lower mitochondrial Ca^2+^ uptake thereby influencing the mitochondrial respiration (Chen et al. 2012b). Interestingly, mitochondria from MFN2 KO hearts are enlarged, have higher Ca^2+^ retention capacity, and show delayed depolarization when subjected to ROS‐induced stress which can all be attributed to the modification jSR–mitochondria interplay (Papanicolaou et al. 2011; Dorn et al. 2015). However, whether mitochondrial–SR‐altered interaction is due to the anatomical changes or other pleiotropic roles of MFN2 was not known. Here, we observed a significant decrease in the number of jSR–mitochondria networks. Consistent with this finding, previous reports have shown structural disorganization of MFN2 KO cardiomyocytes as well as reduction of proteins including RYR2, Serca2a, and phospholamban that reside in this region and facilitate calcium signaling (Papanicolaou et al. 2011; Chen et al. 2012b). These data are in contrast to previous investigations where ablation of MFN2 in mouse embryonic fibroblasts and HeLa cells increased the number of mitochondria–ER contacts (Cosson et al. 2012; Filadi et al. 2015). Furthermore, MFN2 is the key protein in tethering mitochondria and SR, and investigations in mouse embryonic fibroblasts, devoid of MFN2, showed an increase in the distance between the SR and mitochondria (de Brito and Scorrano 2008; Naon et al. 2016). In parallel, our investigation showed for the first time that the anatomical 3D distance between the mitochondria and jSR is increased in the MFN2 KO adult cardiomyocytes. In contrast, previous studies using adult cardiomyocytes have demonstrated no significant increase in the 2D distance between the center of T‐tubule and mitochondria or the transverse side length between the jSR and mitochondria of MFN2 KO and WT mice (Ngoh et al. 2012). Moreover, Cosson et al. (2012) have shown a decrease in the distance between mitochondria and SR in MFN2 KO mouse embryonic fibroblasts. Similarly, acute ablation of MFN2 in HeLa and SH‐SY5Y cells was followed by an increase in the association between ER and mitochondria (Filadi et al. 2015). A possible explanation for these inconsistencies can be the cell‐specific nature of mitochondria–jSR communication as well as differences between acute and chronic models of MFN2 ablation. In addition, 2D quantification may not be sensitive enough to detect changes in the spatial interaction between mitochondrial and SR in 3D.

## Summary

In conclusion, using 3D electron tomography of the adult heart, we have demonstrated that MFN2 deficiency alters mitochondrial 3D shape, reduces the number of mitochondria–jSR networks, and increases the minimum distance between the mitochondria and jSR. These findings confirm the role of MFN2 as a tethering protein between mitochondria and jSR in the adult heart, thereby providing the structural basis for calcium signaling between these two organelles.

## Conflict of Interest

None declared.

## Supporting information




**Figure S1.** Determination of individual mouse genotypes. Animals expressing the Cre gene (marked by white stars) are MFN2^*loxp/loxp*^ KO, whereas those lacking the Cre gene are WT littermate.
**Figure S2.** Distribution of morphometric parameters of mitochondria in WT and MFN2 KO hearts. Box and whiskers plots incorporating the median and 5–95% percentiles of mitochondrial 3D volume and roundness in WT and MFN2 KO are presented in (A) and (B), respectively. Distribution of mitochondrial elongation is shown in (C) and flatness distribution is given in (D); 317 WT and 219 MFN2 KO mitochondria (*N* = 5 WT and MFN2 KO mice).

**Movie S1.** Mitochondria from a KO mouse Jappl.

**Movie S2.** Mitochondria from a WT mouse Jappl.
