# Supplementary material for: Assessing the effects of mitofusin 2 deficiency in the adult heart using 3D electron tomography
Source: Physiol Rep. 2017 Sep 14;5(17):e13437. doi: 10.14814/phy2.13437 (PMC5599868; doi:10.14814/phy2.13437)
Supplement: Supplementary file 1 — Figure S1. Determination of individual mouse genotypes. Animals expressing the Cre gene (marked by white stars) are MFN2loxp/loxp KO, whereas those lacking the Cre gene are WT littermate. Figure S2. Distribution of morphometric parameters of mitochondria in WT and MFN2 KO hearts. Box and whiskers plots incorporating the median and 5–95% percentiles of mitochondrial 3D volume and roundness in WT and MFN2 KO are presented in (A) and (B), respectively. Distribution of mitochondrial elongation is shown in (C) and flatness distribution is given in (D); 317 WT and 219 MFN2 KO mitochondria (N = 5 WT and MFN2 KO mice). [file PHY2-5-e13437-s001.docx]

**Supplementary Data**


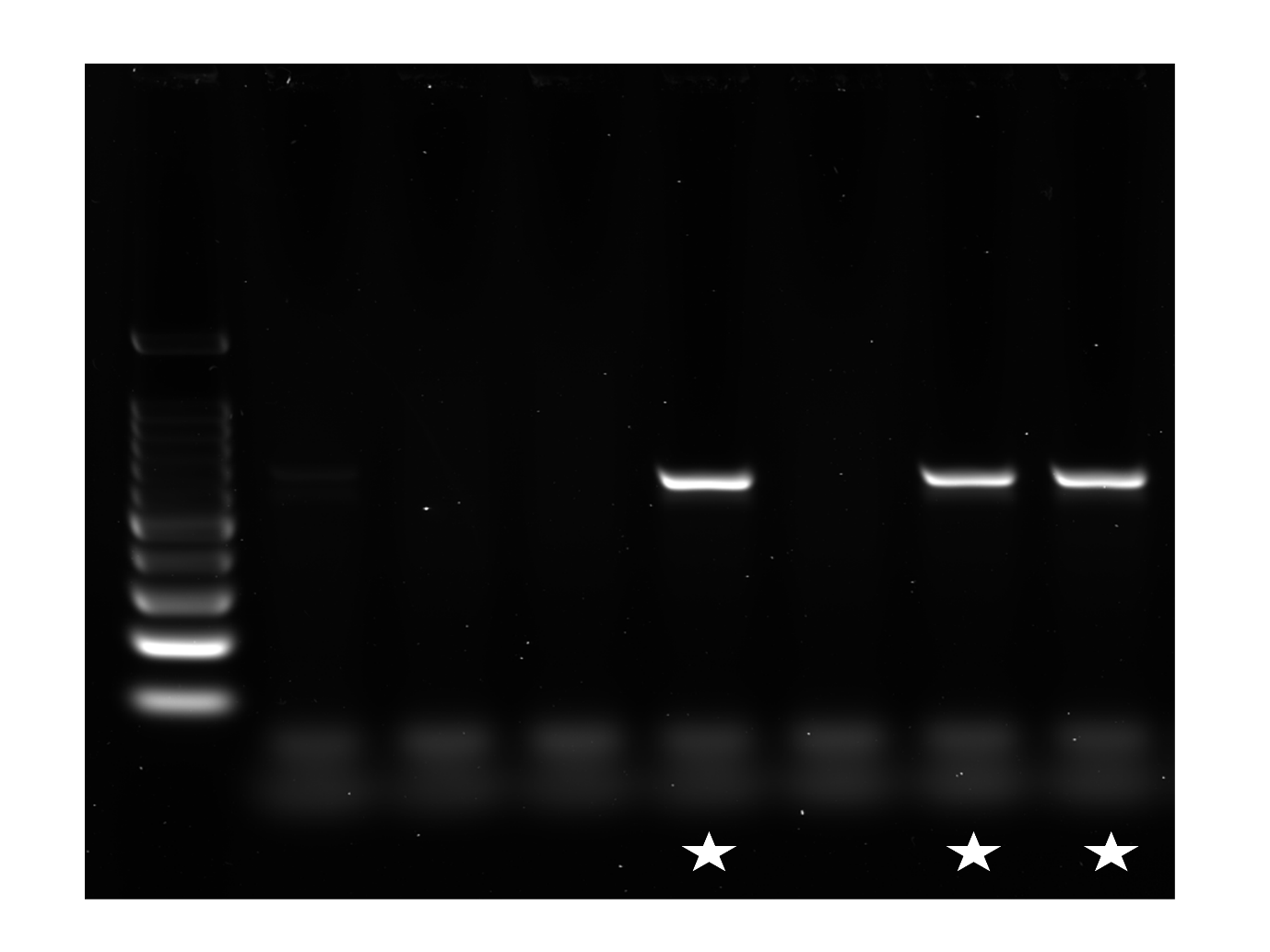


**Supplementary Figure S 1 Determination of individual mouse genotypes.**

Animals expressing the Cre-gene (marked by white stars) are MFN2*^loxp/loxp^* KO whereas those lacking the Cre-gene are WT littermate.


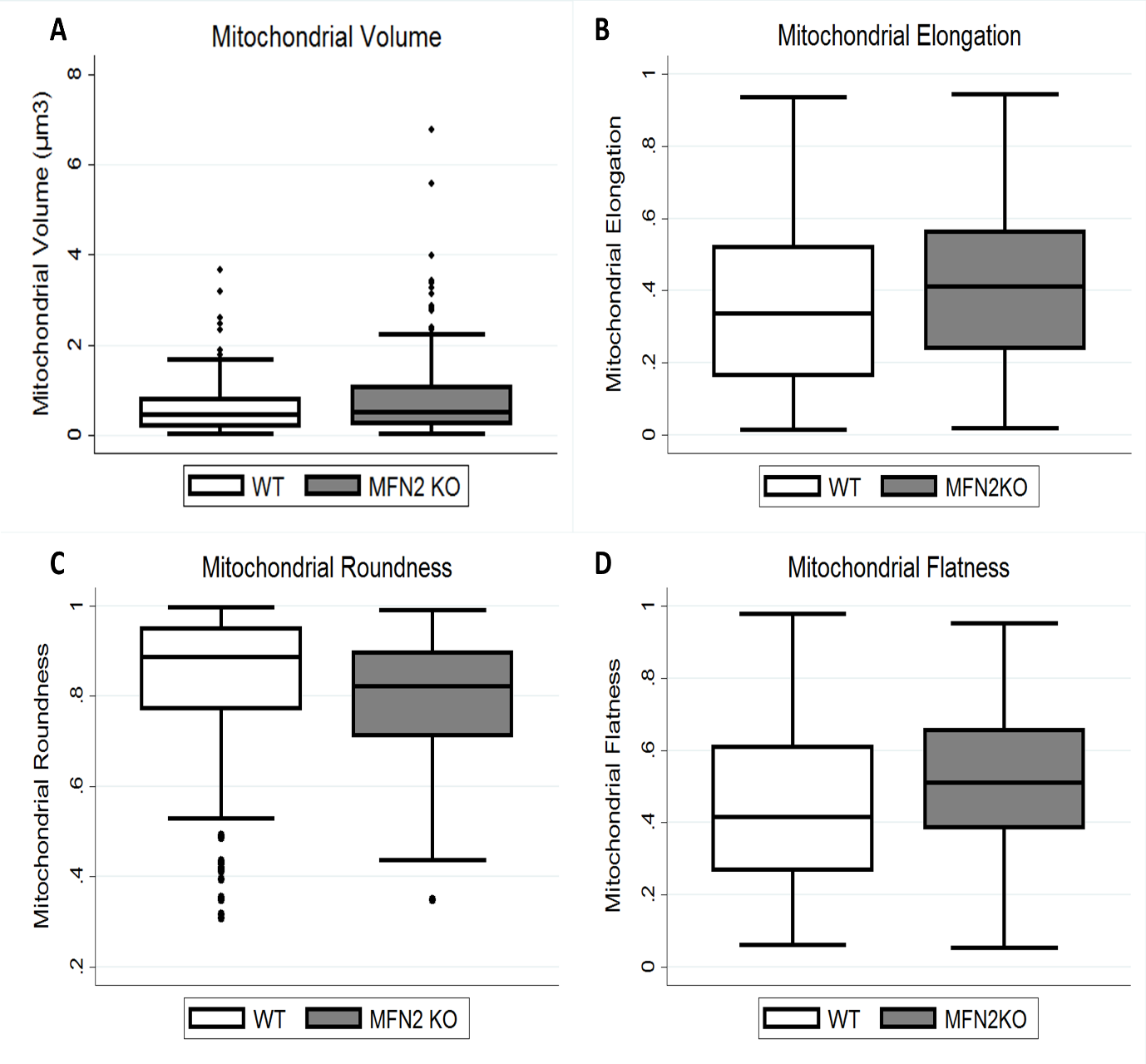


**Supplementary Figure S 2 Distribution of morphometric parameters of mitochondria in WT and MFN2 KO hearts.**

Box and whiskers plots incorporating the median and 5 to 95% percentiles of mitochondrial 3D volume and roundness in WT and MFN2 KO are presented in (A) and (B), respectively. Distribution of mitochondrial elongation is shown in (C) and flatness distribution is given in (D). 317 WT and 219 MFN2 KO mitochondria (N=5 WT and MFN2 KO mice)
